# Explaining unexplained hypoglycemia: How LC-MS/MS can help

**DOI:** 10.1016/j.plabm.2022.e00291

**Published:** 2022-07-12

**Authors:** M.T. Ackermans, J. Hopman, A.C. Heijboer, S.E. Siegelaar

**Affiliations:** a Endocrine Laboratory, Department of Clinical Chemistry, Amsterdam UMC, University of Amsterdam, the Netherlands; b Department of Emergency Medicine, Haaglanden Medical Center, The Hague, the Netherlands; c Endocrine Laboratory, Department of Clinical Chemistry, Amsterdam UMC, Vrije Universiteit Amsterdam, the Netherlands; d Department of Internal Medicine, Endocrinology and Metabolism, Amsterdam UMC, University of Amsterdam, Amsterdam, the Netherlands

## Abstract

Explaining hypoglycaemia, especially in patients without diabetes mellitus, is challenging. Here we present a case, where the added value for clinical diagnosis of insulin determination with liquid chromatography-mass spectrometry (LC-MS/MS) is shown. By the use of LC-MS/MS the different insulin analogues can be identified. The confirmation of an insulin analogue present during hypoglycaemia facilitated in our case the discussion with the patient and his family about what happened.

## 1 Introduction

Hypoglycaemia is a potentially life-threatening condition. Severe hypoglycaemia, defined as a low blood glucose needing third party help for its solution, is a medical emergency that can cause seizure and loss of consciousness and can ultimately result in death.

The differential diagnosis of hypoglycaemia is large, including use of glucose lowering drugs as sulfonylurea, other drugs as, for example, beta-blockers and heart arrhythmia drugs, adrenal insufficiency, critical illness and hyperinsulinaemia [1]. Hyperinsulinaemia can be caused by endogenous insulin production, *i.e.* an insulinoma or use of sulphonylurea as well as an overdose of exogenous insulin. The measurement of serum insulin and c-peptide plays a crucial role in the differential diagnosis of hypoglycaemia. Elevated insulin and c-peptide levels during hypoglycaemia suggest endogenous hyperinsulinaemia, whereas elevated insulin levels and suppressed c-peptide levels are more indicative for exogenous hyperinsulinaemia. Low insulin and low c-peptide levels indicate an origin of the hypoglycaemia independent from insulin action.

Most diagnostic laboratories use automated immunoassays for the measurement of the insulin in serum. The widely prescribed insulin analogues have modifications in their primary amino acid sequence [2]. As a consequence not all commercially available immunoassays will detect insulin analogues in the same way [[3], [4], [5], [6]], which make results difficult to interpret. For example, Parfitt et al. [3] showed that the fast acting insulin analogue Apidra (Sanofi, Paris, France), has a recovery between 1% (AutoDelphia, PerkinElmer, Waltham, MA, USA) and 140% (Iso-Insulin assay, Mercodia, Uppsala, Sweden).

In LC-MS/MS the detection is based on the mass-to-charge (*m*/*z*) ratio of analytes. When the analytes enter the ion source of the mass spectrometer, they are ionized. After the ionization the ions are separated in the first mass analyzer where a specific *m*/*z* ratio can be selected. This so called parent *m*/*z* is guided to a collision cell where it is fragmented. The fragments are then transferred to a second mass analyzer where again a specific *m*/*z* ratio can be selected. This second *m*/*z* ratio is called the daughter ion. The combination of parent/daughter *m*/*z* is analyte specific. Since insulin analogues differ from each other due to modifications in their primary amino acid structure, their molecular mass is different. As a consequence, their parent/daughter *m*/*z* will differ, with the result that LC-MS/MS can make a distinction between these insulin analogues. In the past decade LC-MS/MS methods for insulin and its analogues have been developed [[7], [8], [9]].

Although ample literature is available about the potency of LC-MS/MS to distinguish insulin and its analogues also in diagnostic samples, reports about the added value of these methods in the diagnosis of hypoglycaemia factitia are rare. Here we present a case in which the added value of insulin determination with LC-MS/MS for clinical diagnosis is shown.

## 2 Case presentation

A 72 year-old man was brought in by ambulance to the Emergency Department with decreased consciousness (Glasgow Coma Scale score of 3 out of 15) and snoring breathing. The patient was found on the floor by his roommate. The medical history of the patient included multiple ischemic strokes. The patient used medication for his previous strokes and took 10 mg of zolpidem (benzodiazepine-like hypnotic or ‘sleeping pill’) before bedtime. Patient was feeling well over the past days. Physical examination showed a blood pressure of 150/67 mmHg, saturation 96%, breath frequency 18/min, pulse frequency 90/min and temperature of 35.6 °C. All these values were within the reference range. In the left lower abdomen three small subcutaneous haematomas were observed. Point of care measurement of glucose (Accu-check Inform II, Roche, Basel, Switserland) showed a capillary glucose of 0.5 mmol/L. The hypoglycaemia was later confirmed in the laboratory (GLUC3 Enzymatic reference method, Roche, Basel Switserland). The appearance of an unconscious patient, small subcutaneous hematoma's in combination with the low blood glucose level raised suspicion of hypoglycemia factitia by administration of exogenous insulin. Treatment was initiated with 50 mL of 50% intravenous glucose followed by a continuous infusion of 2L/24h 20% glucose and blood glucose levels were checked repeatedly resulting in significant improvement to a blood glucose above the hypoglycaemic range within 1 h (Table 1). The patient told us he generally administers the fast acting insulin analogue Apidra (Sanofi-Aventis, Paris, France) and the long acting insulin analogue Lantus (Sanofi-Aventis, Paris, France) to his roommate, who was unable to do it herself, as he did the evening before. He denied he had injected insulin to himself and denied a suicidal attempt.

**Table 1:** Results of laboratory analysis from the patient.

	Assay*	Reference value	7.02 hour	7.24 hour	08.00 hour	8.34 hour
Glucose (mmol/L)	GLUC3	3.5–7.8	0.4	29.0	17.5	3.9
Cortisol (nmol/L)	Elecsys cortisol II	170–500	330			
Paracetamol (mg/L)	ACET2	<3.0	<3.0			
C-peptide (nmol/L)	Elecsys c-peptide	0.37–1.5	0.073			
Insulin (mU/L)	Elecsys insulin	2.6–4.9	33.4			

*All assays are measured on Roche systems (Roche, Basel, Switserland).

**Fig. 1:**
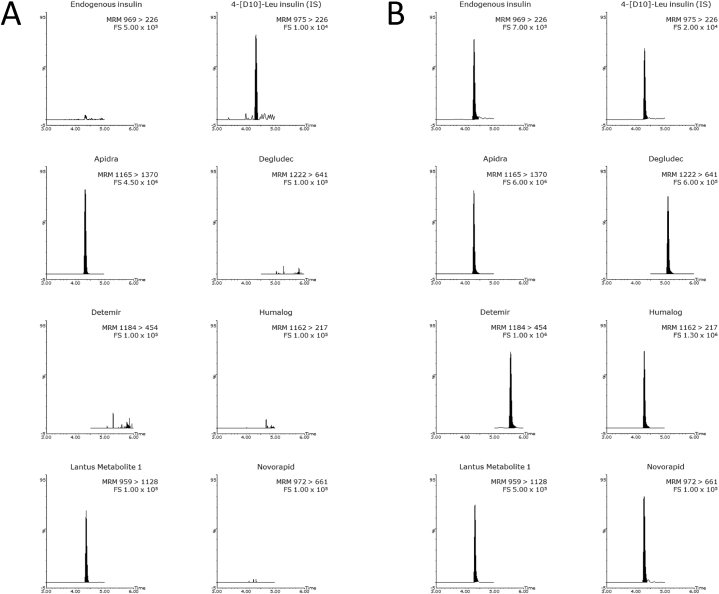
LS-MS/MS chromatograms of (A) the case patient sample and (B) positive control. The panels show the chromatograms for the different insulins. The insulins can be identified based upon their retention time and specific MRM transition.

The patient was hospitalised on a medical psychiatric unit because of high suspicion of hypoglycaemia factitia. Regular laboratory analysis using immunoassays showed a high insulin and low c-peptide level, suggesting an exogenous source of the insulin (Table 1). Further examination using LC-MS/MS confirmed the presence of exogenous insulins, both Apidra and Lantus (see Fig. 1A). As part of the differential diagnosis of hypoglycaemia alcohol intoxication, paracetamol intoxication and adrenal cortex insufficiency were assessed and ruled out (Table 1). During hospital stay blood glucose levels were stable and psychiatric evaluation showed no depression or suicidal thoughts. Most likely, the patient had injected himself with his roommates insulin. Possibly the intake of zolpidem has caused more severe sedation as well as confusion than expected in this person. They were advised starting home care for safe insulin administration to the roommate. After this intervention, no further problems occurred.

## 3 Laboratory tests

### 3.1 Analytical methods

Glucose, cortisol, c-peptide, and insulin were measured using an automated immunoassay (Cobas 6000, Roche Diagnostics, Rotkreuz, Switzerland).

In order to be able to make a distinction between endogenous insulin and the different insulin analogues an in house LC-MS/MS method was used which was based on the methods as described previously by Peterman [9] and Chambers [10]. Briefly, 250 μL internal standard (4-[D-10Leu] insulin, Peptides International, Louisville, KY, USA) in 150 mM octyl-β-glucoside in PBS and 250 μL positive control or sample were pipetted into a 96 well low protein bind plate (Thermo Scientific, Waltham, MA, USA). The insulin in the sample was extracted by immuno-extraction using the MSIA D.A.R.Ts (Thermo Scientific, Waltham, MA, USA) (see Supplemental Table S1). LC-MS/MS was used to separate and determine the different insulin analogues. The separation was achieved on a Cortex UPLC C18+ column (Waters, Milford, MA, USA) using gradient elution. The LC system was coupled to a Xevo-TQ-S, triple quadruple mass spectrometer (Waters, Milford, MA, USA). Detailed analytical parameters are given in Supplemental Tables S2- S4.The analytical performance characteristics of the method are given in Supplemental Table S5 and Supplemental Fig. 1. The duration of the assay is about 4 hours.

A positive control was prepared containing all the insulin analogues at a concentration of 200 pmol/L (34 mU/L) from the 100 IU/mL ampoules of insulin analogues (see Supplemental information for details of the chemicals), the high calibrator of the Atellica insulin assay (Siemens-Healthineers, Den Haag, The Netherlands) and the Glargine Metabolite 1 (provided by Sanofi-Aventis). The positive control was kept at −80 °C until analysis. Fig. 1B shows a representative chromatogram of this positive control.

### 3.2 Patient consent

The patient was informed about this case presentation and gave informed consent to use the data.

## 4 Discussion

Today, most diagnostic laboratories use automated inmmunoassay analyzers to determine insulin concentrations in patient samples. In immunoassays, the antibody recognizes an epitope on the analyte of interest. If the same epitope is present in another analyte, this will result in so-called cross-reactivity. This might be the case for the insulin analogues. The extent to which an immunoassay determines an insulin analogues depends upon the antibody used.

On the other hand, in LC-MS/MS the determination of insulin and its analogues is based on the molecular weight. Most insulin analogues differ in molecular weight from endogenous insulin and each other. Therefore, although both immunoassays and LC-MS/MS based methods can be used to determine insulin, only LC-MS/MS based methods make it possible to detect different insulin analogues separately and interpret the results without ambiguity [4].

In our case patient the hypoglycaemia was confirmed by the Roche immunoassay. With the in-house LC-MS/MS method we could determine the cause of this hypoglycaemia and confirm hypoglycaemia factitia. As a result appropriate measures were taken to prevent future problems. In the absence of sulfonylurea use, measurement of high insulin and low c-peptide concentrations during hypoglycaemia is suggestive of exogenous insulin administration. However, the possibility of naming the specific insulin analogue used, strengthens the suspicion of the physician and facilitates the discussion with the patient. In other cases with both low insulin and low c-peptide concentrations, due to low cross reactivity of the insulin analogue in the immunoassay, it might be crucial to measure insulin analogues using LC-MS/MS to either demonstrate or exclude hypoglycaemia factitia.

## 5 Conclusion

The presence of hypoglycaemia in a patient without diabetes mellitus is a challenging finding. In the differential diagnosis the insulin and c-peptide concentrations during hypoglycaemia are key measurements to determine to the true diagnosis. Qualitative measurement of the insulin analogue present during hypoglycaemia is possible and facilitated in our case the discussion with the patient and his family about what happened.

## Declaration of competing interest

None.
