# Supplementary material for: Explaining unexplained hypoglycemia: How LC-MS/MS can help
Source: Pract Lab Med. 2022 Jul 12;31:e00291. doi: 10.1016/j.plabm.2022.e00291 (PMC9289730; doi:10.1016/j.plabm.2022.e00291)
Supplement: Multimedia component 1 [file mmc1.docx]

**Supplemental data:**

**Chemicals and materials**

Acetonitrile (ACN, art.nr. 1207801) and formic acid (art.nr 6914143) were purchased from Biosolve (Valkenswaard, The Netherlands). Leucine enkephaline (art.nr L9133) and octyl-β-glucoside (art.nr. 850511P) were purchased from Sigma Aldrich (Zwijndrecht, The Netherlands). MSIA D.A.R.T (art nr 991001096) and tri-fluoroacetic acid (TFA) were purchased from Thermo Scientific (Waltham, MA, USA). Phosphate buffered saline (PBS, art.nr 13763806) was purchased from Fresenius Kabi (Huis ter Heide, The Netherlands). The internal standard (IS) 4-[D10-leu] insulin (art.nr PLP-3404-V) was purchased from Peptides International (Louisville, KY, USA). Insulin glulisine (Apidra), and insulin glargine (Lantus) were purchased from Sanofi-Aventis (Paris, France). Insulin detemir (Levemir), insulin aspart (Novorapid), human insulin (Actrapid) and insulin tresiba (Degludec) were purchased from Novo-Nordisk (Bagsværd, Denmark). Insulin Lispro (Humalog) was purchased from Eli Lilly (Indianapolis, IN, USA). All insulin analogs were purchased at a concentration of 100 IU/mL. Lantus Metabolite 1 was kindly provided by Sanofi-Aventis (Frankfurt am Main, Germany). Calibrators for the Atellica insulin assay (art.nr 1099562) were purchased from Siemens (Siemens-Healthineers, Den Haag, The Netherlands). UPLC grade water was delivered by a Pure Flex system from Elga (Veolia Water Solutions & Technologies, Ede, The Netherlands).

**S1**: **MSIA D.A.R.T. protocol**

| **Step** | **Volume (uL)** | **Speed** | **Cycles** |
| --- | --- | --- | --- |
| Wash (PBS) | 150 | 4 | 20 |
| Capture | 250 | 1 | 500 |
| Wash (PBS) | 150 | 4 | 20 |
| Wash (PBS) | 150 | 4 | 20 |
| Wash (H_2_O) | 150 | 4 | 20 |
| Wash (H_2_O) | 150 | 4 | 20 |
| Elute (33% ACN, 0.4% TFA, 190 ug/mL Leucine Enkephaline) | 50 | 1 | 100 |

**S2**: **LC instrument parameters**

| Instrument | Waters Acquity UPLC system |
| --- | --- |
| Pump | Binary Solvent Manager |
| Column | Waters Cortecs UPLC C18+ |
| Column temperature (˚C) | 60 |
| Column dimensions | 50 x 2.1 mm, 1.7 µm |
| Mobile Phase A | H_2_O + 0.1% formic acid |
| Mobile Phase B | Acetonitrile + 0.1% formic acid |
| Flow (mL/min) | 0.250 |
| Gradient | \| Time (min) \| %B \| Curve \| \| --- \| --- \| --- \| \| Initial \| 15 \| Initial \| \| 2.00 \| 15 \| 6 \| \| 5.00 \| 40 \| 6 \| \| 5.10 \| 90 \| 6 \| \| 6.10 \| 90 \| 6 \| \| 6.50 \| 15 \| 6 \| \| 8.50 \| 15 \| 6 \| |
| Injector | Fixed Loop |
| Injection volume (µL) | 25 |

**S3**: **Waters Xevo-TQ-S** **MS instrument parameters**

| Source | Ion mode | ESI+ |
| --- | --- | --- |
|  | Capillary voltage | 2.5 kV |
|  | Source offset | 50 V |
|  | Source temperature | 150 ˚C |
|  | Desolvation temperature | 600 ˚C |
|  | Desolvation gas flow (N_2_) | 1000 L/hr |
|  | Cone gas flow (N_2_) | 150 L/hr |
| Analyzer 1 | LM resolution | 2.5 |
|  | HM resolution | 13.5 |
|  | Ion energy 1 | 0.2 |
| Collision cell | Collision gas (Ar) | 0.15 mL/min |
|  | Entrance | 1 |
|  | Collision energy | Variable |
|  | Exit | 1 |
| Analyzer 1 | LM resolution | 2.5 |
|  | HM resolution | 13.5 |
|  | Ion energy 2 | 0.8 |

**S4: MRM parameters**

| **Component** | | **MRM** | **Cone voltage (V)** | **Collision energy (eV)** |
| --- | --- | --- | --- | --- |
| **Endogenous insulin** | |  |  |  |
|  | Quantifier | 968.7>226.2 | 35 | 45 |
|  | Qualifier | 1162.0>226.2 | 35 | 45 |
| **4-[D10-Leu] insulin (IS)** | |  |  |  |
|  | Quantifier | 975.0>226.0 | 40 | 30 |
|  | Qualifier | 1170.0>226.0 | 60 | 30 |
| **Apidra** | |  |  |  |
|  | Quantifier | 1165.4>1370.0 | 40 | 22 |
|  | Qualifier | 1165.4>346.2 | 14 | 22 |
| **Degludec** | |  |  |  |
|  | Quantifier | 1221.6>641.4 | 20 | 25 |
|  | Qualifier | 1018.2>1093.2 | 20 | 15 |
| **Detemir** | |  |  |  |
|  | Quantifier | 1184.2>454.5 | 40 | 28 |
|  | Qualifier | 1184.1>1366.7 | 40 | 22 |
| **Humalog** | |  |  |  |
|  | Quantifier | 1162.3>217.2 | 50 | 40 |
|  | Qualifier | 968.2>217.0 | 50 | 40 |
| **Lantus Metabolite 1** | |  |  |  |
|  | Quantifier | 959.4>1128.4 | 20 | 20 |
|  | Qualifier | 959.4>1118.4 | 20 | 21 |
| **Novorapid** | |  |  |  |
|  | Quantifier | 971.8>660.9 | 50 | 18 |
|  | Qualifier | 971.8>1139.4 | 12 | 18 |

**S5: Analytical performance characteristics**

|  | | **Novorapid** | **Apidra** | **Humalog** | **Detemir** | **Degludec** | **Lantus M1** |
| --- | --- | --- | --- | --- | --- | --- | --- |
| **Accuracy (%)** | | 95 | 97 | 102 | 109 | 74 | 103 |
| **Precision (%)** | |  |  |  |  |  |  |
|  | Concentration  (range 15-1200 pmol/L) | 12 | 8 | 11 | 25 | 21 | 18 |
|  | 1^st^ target ratio | 6 | 4 | 8 | 6 | 27 | 5 |
| **Carry-over (%)** | | 0.4 | 0.6 | 0.3 | 1.5 | 2.1 | 0.4 |

**Supplemental Figure 1:**

Passing and Bablok regression of the LC-MS/MS method versus the Atellica insulin assay (Siemens-Healthineers, Den Haag, The Netherlands) for the determination of endogenous insulin using left-over patient samples (N=10).

**

R^2^=0.979
